# Long-term glucose-lowering effect of intermittently scanned continuous glucose monitoring for type 1 diabetes patients in poor glycaemic control from Region North Denmark: An observational real-world cohort study

**DOI:** 10.1371/journal.pone.0274626

**Published:** 2022-10-14

**Authors:** Morten Hasselstrøm Jensen, Simon Lebech Cichosz, Peter Gustenhoff, Amar Nikontovic, Ole Hejlesen, Peter Vestergaard

**Affiliations:** 1 Steno Diabetes Center North Denmark, Aalborg University Hospital, Aalborg, Denmark; 2 Department of Health Science and Technology, Aalborg University, Aalborg, Denmark; 3 Department of Endocrinology, Aalborg University Hospital, Aalborg, Denmark; 4 Department of Clinical Medicine, Aalborg University Hospital, Aalborg, Denmark; Ospedale San Raffaele, ITALY

## Abstract

**Background:**

Lowering glucose levels is a complex task for patients with type 1 diabetes, and they often lack contact with health care professionals. Intermittently scanned continuous glucose monitoring (isCGM) has the potential to aid them with blood glucose management at home. The aim of this study was to investigate the long-term effect of isCGM on HbA_1c_ in type 1 diabetes patients with poor glycaemic control in a region-wide real-world setting.

**Methods:**

All patients with type 1 diabetes receiving an isCGM due to poor glycaemic control (≥70 mmol/mol [≥8.6%]) in the period of 2020–21 in Region North Denmark (“T1D-CGM”) were compared with all type 1 diabetes patients without isCGM (“T1D-NOCGM”) in the same period. A multiple linear regression model adjusted for age, sex, diabetes duration and use of continuous subcutaneous insulin infusion was constructed to estimate the difference in change from baseline HbA_1c_ between the two groups and within subgroups of T1D-CGM.

**Results:**

A total of 2,527 patients (T1D-CGM: 897; T1D-NOCGM: 1,630) were included in the study. The estimated adjusted difference in change from baseline HbA_1c_ between T1D-CGM vs T1D-NOCGM was -5.68 mmol/mol (95% CI: (-6.69 to -4.67 mmol/mol; p<0.0001)). Older patients using isCGM dropped less in HbA_1c_.

**Conclusions:**

Our results indicate that patients with type 1 diabetes in poor glycaemic control from Region North Denmark in general benefit from using isCGM with a sustained 24-month improvement in HbA_1c_, but the effect on HbA_1c_ may be less pronounced for older patients.

## Introduction

Lowering glucose levels is not a trivial task for patients with type 1 diabetes (T1D) and includes a complex interplay between several factors in situations without close contact with health care professionals [1, 2]. Several studies have indicated that visualization of interstitial glucose from intermittently scanned continuous glucose monitoring (isCGM) leads to optimized glycaemic control by providing the users with a deeper insight into their own glucose metabolism unveiling some of the factors’ interplay [3–11]. Furthermore, the addition of real-time alarms in newer isCGM devices might lead to further improvement [12]. However, these studies are typically of a shorter duration of 3–6 months and with strict inclusion/exclusion criteria [13–15]. It is suspected that digital interventions, such as isCGM, have a great impact on glycaemic control in the first half year with a less pronounced or even no effect at later stages, and studies with longer durations in real-world settings without close contact with health care professionals are therefore needed [16]. Furthermore, several indications for the use of isCGM are sometimes present in the patient groups under investigation, which may blur glucose-lowering treatment effects [17]. To sum up, we hypothesized that the effect of isCGM in patients with T1D is greatest in the first half year and is thereafter reduced. To assess the real-world long-term effect of isCGM including both devices with and without alarms is novel and a needed research study for the benefit of patients, clinicians, and decision makers.

The aim of this study was to investigate the long-term effect of isCGM on HbA_1c_ in patients with T1D in a region-wide real-world setting.

## Methods

### Study design

This was a region-wide real-world cohort study of T1D patients receiving an isCGM device (Freestyle Libre 1 or 2, Abbott Laboratories, Illinois, US) due to poor glycaemic control in the period of 2020–21 in Region North Denmark. isCGM devices for patients with T1D are reimbursed based on nationally defined indications, such as poor glycaemic control (HbA_1c_ ≥ 70 mmol/mol [≥ 8.6%]), pregnancy and hypoglycaemia unawareness, but the focus in this study was patients with poor glycaemic control. The baseline period was defined as the period 12 months before to the date of receiving the isCGM (the index date). The inclusion criteria were adults (≥19 years old at index date) with at least one HbA_1c_ value above 70 mmol/mol in the baseline period to exclude patients receiving isCGM for other indications. All patients with T1D receiving an isCGM (“T1D-CGM”) were compared with all T1D patients who did not receive an isCGM device (“T1D-NOCGM”) with respect to HbA_1c_. CGM metrics were not available. Patients in the T1D-NOCGM group inherited the date of initiation of isCGM as an index date from the T1D-CGM patient with the closest year of birth.

### Education

In Region North Denmark, patients receive education in the use of isCGM. At the first outpatient visit at the Department of Endocrinology, patients in groups of eight receive the isCGMs and receive a 2-hour training program with diabetes nurses on how to use the device and how to interpret the data. Then, a half-hour individual session is held with a diabetes nurse. Three months after, an outpatient visit with a similar structure follows for the same group of patients. Between visits, patients are encouraged to contact the Department of Endocrinology in case of questions or technological challenges.

### Follow-up and outcome

Patients in the cohort were followed from 12 months before the index date to 24 months after in timepoints of quarterly intervals: -12, -9, -6,…,18, 21, 24. HbA_1c_ values were extracted in the 3-year follow-up period, and each value was carried forward to the nearest timepoint. For example, an HbA_1c_ value 45 days after the index date was carried forward to month 3. If a patient had multiple HbA_1c_ values within a quarter the average was carried forward. The outcome of the study was change from baseline in HbA_1c_. Baseline HbA_1c_ for a patient was calculated as the average of all HbA_1c_ values for the given patient within the baseline period. This baseline HbA_1c_ was chosen instead of using the value at the index date to reduce bias from randomly high HbA_1c_ levels at the time of consideration of isCGM as treatment intensification by the diabetes specialist.

### Statistical analysis

Descriptive statistics are presented as means with standard deviations (SDs) or counts and percentages of patients. Mann–Whitney U tests, t tests and χ^2^ tests were used to present statistical differences in patient characteristics. Age and diabetes duration were calculated from the date of birth and the date of diabetes diagnosis, respectively, to the index date. Mean and standard error of the mean of change from baseline HbA_1c_ are presented for T1D-CGM vs T1D-NOCGM in a graph together with statistical significance notation derived from a multiple linear regression model of change from baseline HbA_1c_ as dependent variable and timepoints as a categorical independent variable. The reference was the baseline HbA_1c_ added as a dummy timepoint. Another change from baseline was calculated as the difference between baseline HbA_1c_ and the mean of all HbA_1c_ values after the index date. The mean and 95% confidence interval for this change from baseline HbA_1c_ are presented in a table for T1D-CGM and T1D-NOCGM together with p-values from t-tests. Furthermore, the adjusted difference between this change from baseline for T1D-CGM versus T1D-NOCGM is shown and was derived from a multiple linear regression model adjusted for sex, age, diabetes duration and use of continuous subcutaneous insulin infusion (CSII), and the analysis was repeated within the T1D-CGM group for dichotomous stratifications of HbA_1c_ at baseline, age, sex, and diabetes duration. The stratification cutoff for the numeric variables was objectively defined as mean + SD of the variable (rounded to the nearest whole number).

All analyses were conducted in SAS 9.4 (SAS Institute Inc., Cary, North Carolina, US).

### Data sources

Data were extracted from the Region North Denmark Diabetes Dataplatform. The Diabetes Dataplatform is a real-time quality and research database collecting data from multiple hospital systems, including demographics, laboratory, and prescription data for all patients with diabetes who have had any contact with the hospitals in Region North Denmark. HbA_1c_ values were extracted from the local laboratories where blood samples undergo state-of-the-art analysis. Blood sampling was performed by diabetes nurses, lab technicians or endocrinologists at the patients’ outpatient visits, The validity of the HbA1c values in this study is therefore deemed high.

### Ethics

The study was approved by the local executive management at Aalborg University Hospital and North Denmark Regional Hospital. Participant consent was not required, because this investigation was part of quality assurance project and data were analyzed anonymously.

## Results

**Fig 1:**
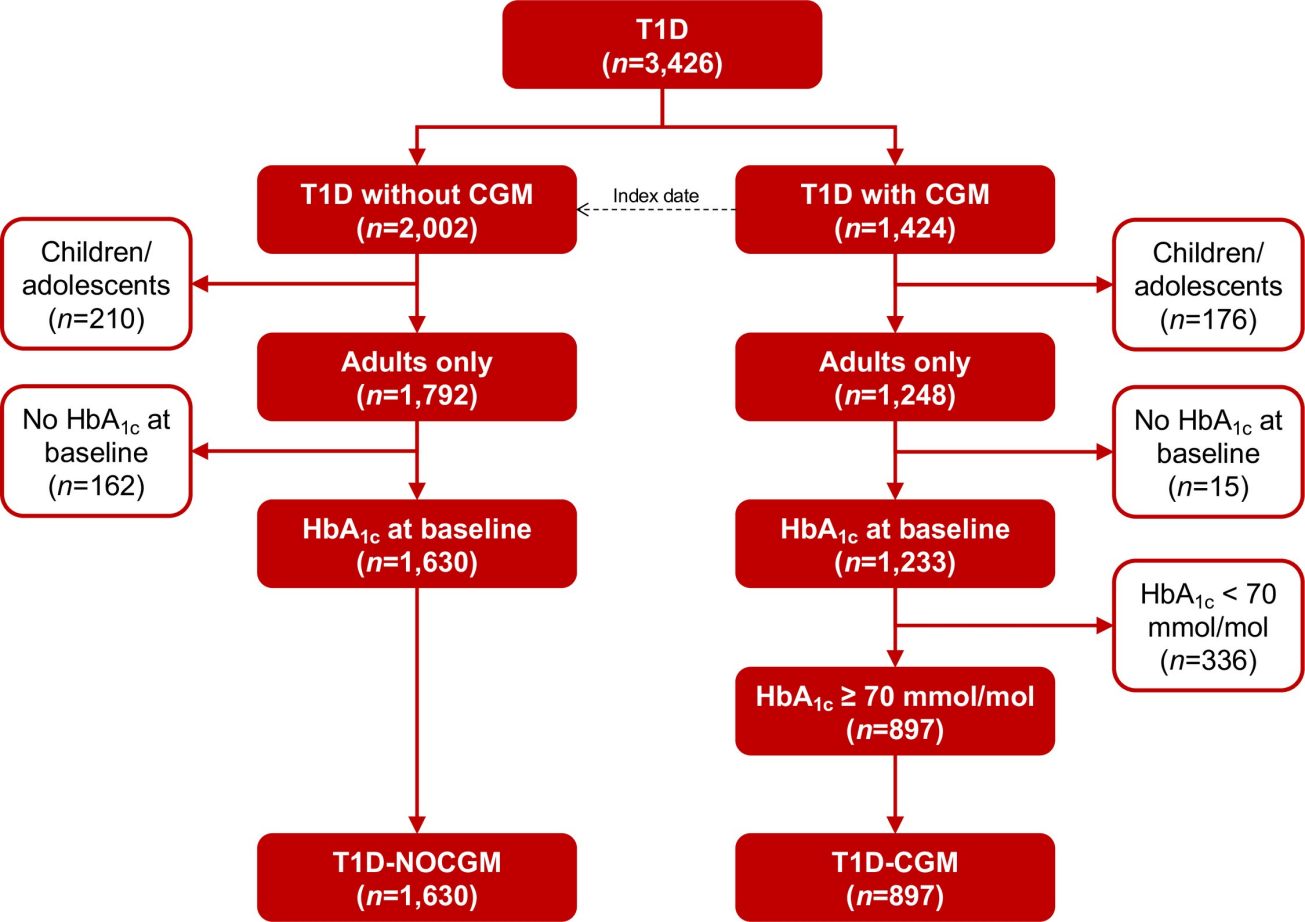
The number of patients with T1D included in the study.

**Fig 2:**
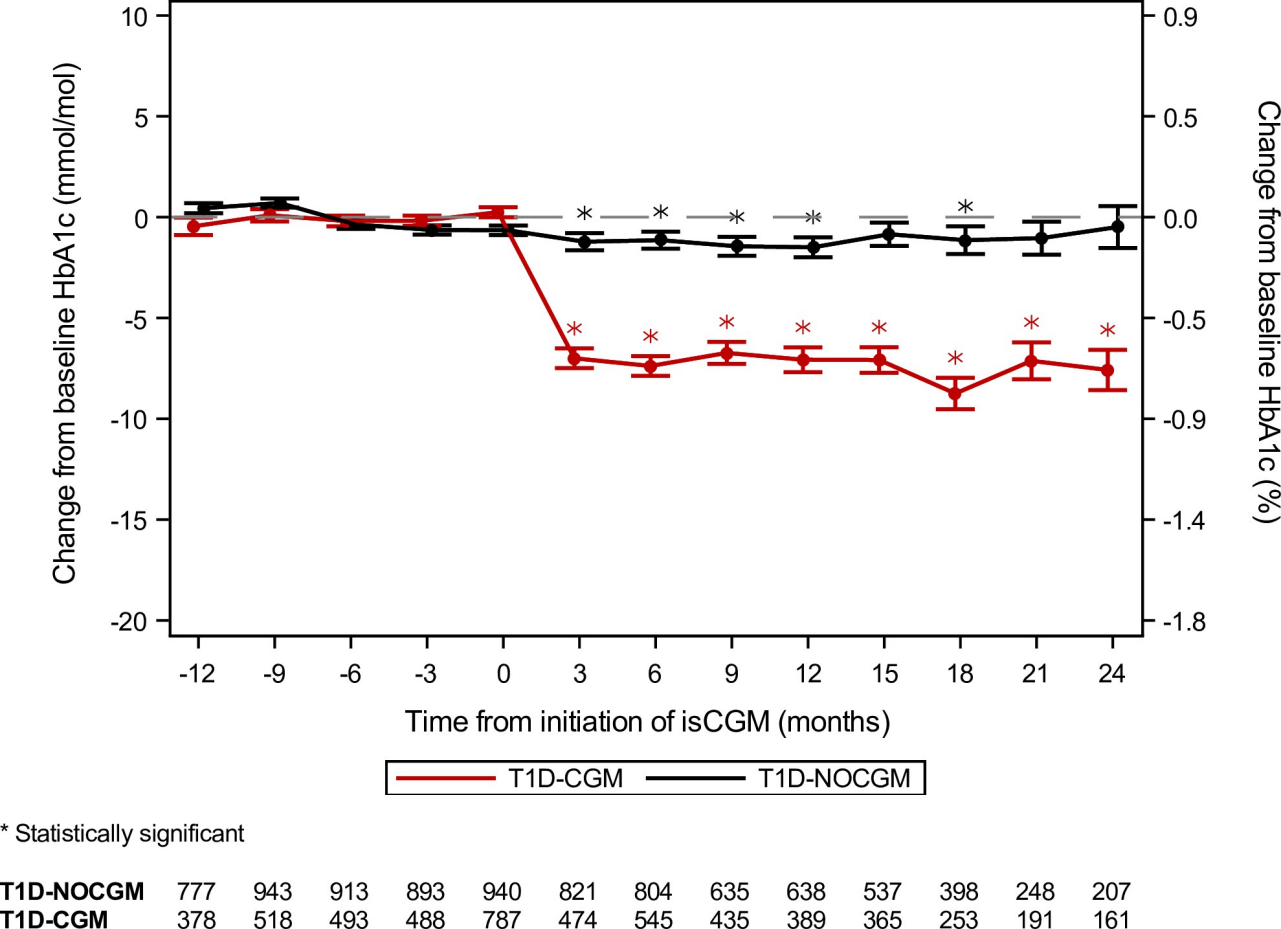
Observed mean and standard error of the mean of change from baseline HbA_1c_ for T1D-CGM vs T1D-NOCGM. The statistically significant asterisk notation is from a multiple linear regression model with change from baseline HbA_1c_ as the dependent variable, time as the independent variable and HbA_1c_ at baseline as the reference, i.e. the statistical significant notation refers to baseline within the group. Available HbA_1c_ values in the quarter before each time point contributed to the means.

**Fig 3:**
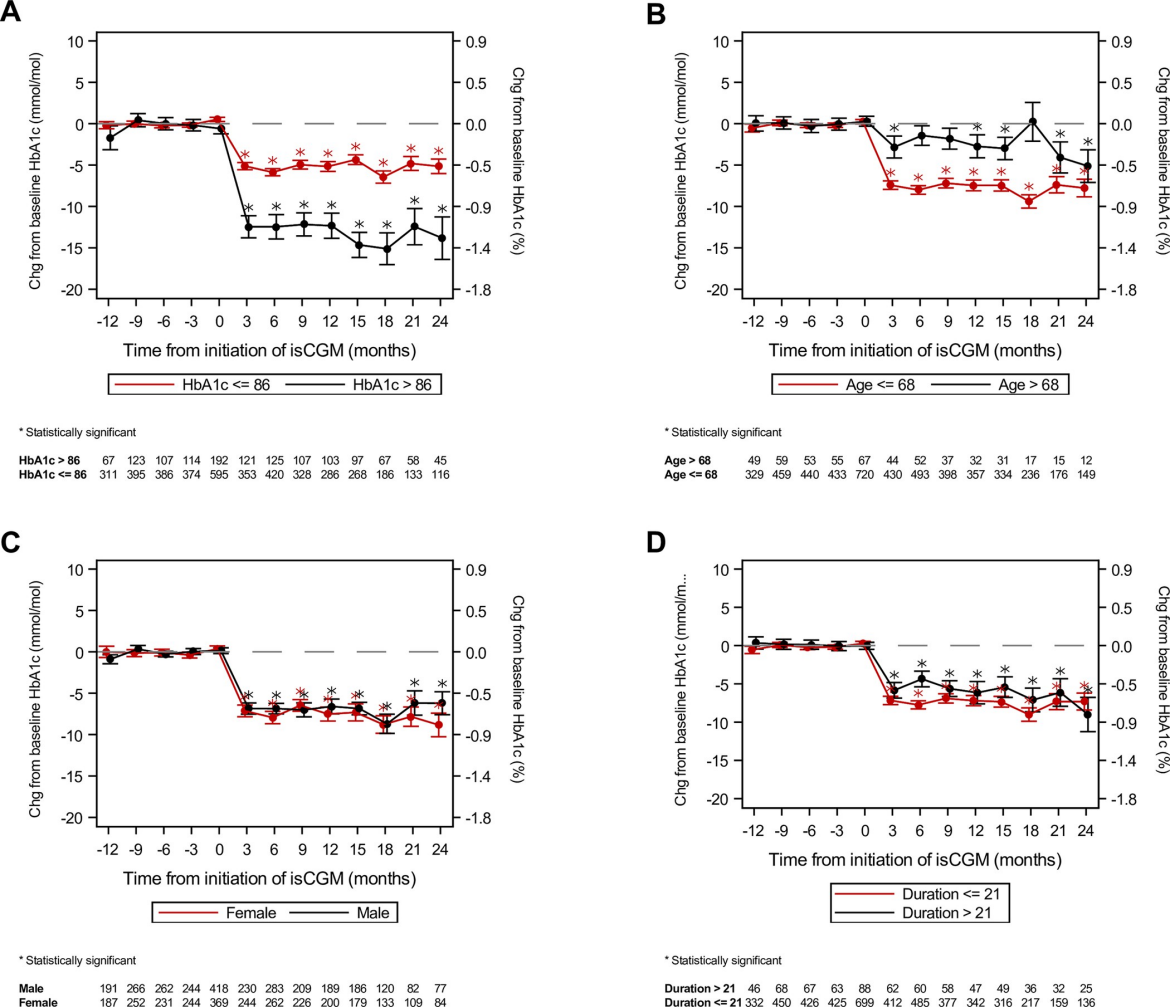
Mean and standard error of the mean of change from baseline HbA_1c_ for subgroups in the T1D-CGM group. The upper left graph (Fig 3A) shows patients with HbA_1c_ ≤ 86 mmol/mol at baseline versus patients with HbA_1c_ > 86 mmol/mol at baseline. The upper right graph (Fig 3B) shows patients aged ≤ 68 years versus patients aged > 68 years. The bottom left graph (Fig 3C) is females versus males. The bottom right graph (Fig 3D) shows patients with a diabetes duration ≤ 21 years versus patients with a diabetes duration > 21 years. The estimates were derived from general linear models with change from baseline in HbA_1c_ as the dependent variable, time as the independent variable and HbA_1c_ at baseline as the reference. Available HbA_1c_ values in the quarter before each time point contributed to the estimates. Statistically significant changes are marked with an asterisk and refers to baseline within the group.

In total, 3,426 people had T1D in the Region North Denmark, and 1,424 received an isCGM, see Fig 1. After applying the inclusion criteria, the final cohort consisted of 2,527 patients with T1D (T1D-CGM: 897; T1D-NOCGM: 1,639). The baseline characteristics of the final cohort are shown in Table 1. In Table 2, the mean change in baseline HbA_1c_ and number of hospital visits can be seen for T1D-CGM and T1D-NOCGM. Fig 2 shows the mean change in baseline HbA_1c_ for T1D-CGM and T1D-NOCGM during the three years follow-up, and Fig 3 shows the mean change in baseline HbA_1c_ for subgroups within the T1D-CGM group in the follow-up period. Table 3 shows the estimated adjusted difference in change from baseline HbA_1c_ between T1D-CGM and T1D-NOCGM and between subgroups within T1D-CGM. In Fig 2, HbA_1c_ levels drop with approx. 7 mmol/mol after receiving an isCGM, and the drop remains fairly stable for the following 24 months. In Fig 3, baseline HbA_1c_ level is one of the most clinically significant predictors of the effect of isCGM, and the effect on patients with baseline HbA_1c_ above 86 mmol/mol is greater than for patients with baseline HbA_1c_ below 86 mmol/mol. Age is also a clinically significant predictor where isCGM for patients above 67 years seems to have a small effect on HbA_1c_.

**Table 1:** Baseline characteristics of the cohort.

	T1D-CGM	T1D-NOCGM	*p* value
Number of individuals	897	1,630	
Age (yrs), mean (SD)	45.5 (15.7)	53.9 (16.7)	< .0001
Age category (yrs), n (%)			
18–29	180 (20.1)	187 (11.5)	< .0001
30–39	150 (16.7)	146 (9.0)	< .0001
40–49	177 (19.7)	291 (17.9)	0.2445
50–59	202 (22.5)	320 (19.6)	0.0862
60–69	121 (13.5)	351 (21.5)	< .0001
70–79	56 (6.2)	253 (15.5)	< .0001
≥80	11 (1.2)	82 (5.0)	< .0001
Sex, n (%)			
Female	422 (47.0)	667 (40.9)	0.0029
Male	475 (53.0)	963 (59.1)	0.0029
HbA_1c_ (mmol/mol), mean (SD)	79.5 (11.9)	64.1 (15.9)	< .0001
Diabetes duration (yrs), mean (SD)	11.6 (8.1)	11.5 (10.4)	< .0001
CSII, n (%)	206 (23.0)	189 (11.6)	< .0001
Late-diabetic complications, n (%)			
Retinopathy	83 (30.3)	140 (28.2)	0.6091
Neuropathy	38 (13.9)	77 (15.5)	0.5967
Nephropathy	5 (1.8)	12 (2.4)	0.6027
Multiple	70 (25.5)	126 (25.4)	0.9478
Other	78 (28.5)	142 (28.6)	0.9944

**Table 2:** Mean, 95% confidence interval (95% CI) and p values for change from baseline HbA_1c_ and for number of hospital visits per year for T1D-CGM and T1D-NOCGM. Estimates were derived from t tests. p value for hospital visits is for test of difference between groups, whereas p values for change from baseline HbA1c is difference before and after baseline within each group.

	Mean (95% CI)	p value
*Change from baseline HbA*_*1c*_		
T1D-CGM	-7.09 (-7.82; -6.36) mmol/mol	< .0001
	-0.65 (-0.72; -0.58) %	
T1D-NOCGM	-0.95 (-1.56; -0.34) mmol/mol	0.0025
	-0.09 (-0.14; -0.03) %	
*Number of hospital visits per year*		
T1D-CGM	3.62 (2.34; 4.90)	0.3266
T1D-NOCGM	3.00 (2.42; 3.59)	

**Table 3:** Adjusted differences in change from baseline HbA_1c_ (mmol/mol) between T1D-CGM and T1D-NOCGM and within the T1D-CGM subgroups of age, sex, HbA_1c_ at baseline and diabetes duration. Estimates and p values are from multiple linear regression models adjusted for age, sex, diabetes duration and use of CSII (Y/N).

	Estimate (95% CI)	p value
*Adjusted difference in change from baseline HbA*_*1c*_		
T1D-CGM–T1D-NOCGM	-5.68 (-6.69; -4.67) mmol/mol	< .0001
	-0.52 (-0.61; -0.43) %	
*Adjusted difference in change from baseline HbA*_*1c*_ *within T1D-CGM subgroups*		
Age	1.40 (-0.27; 3.07) mmol/mol	0.1005
(“>68 yrs”–“≤68 yrs”)	0.13 (-0.02; 0.28) %	
Sex	-0.93 (-1.91; 0.05) mmol/mol	0.0630
(Female–Male)	-0.09 (-0.18; 0.00) %	
HbA1c at baseline	-13.1 (-14.4; -11.8) mmol/mol	< .0001
(“>86 mmol/mol”–“≤86 mmol/mol”)	-1.20 (-1.32; -1.08) %	
Diabetes duration	-1.00 (-3.28; 1.29) mmol/mol	0.3916
(“>21 yrs”–“≤21 yrs”)	-0.09 (-0.30; 0.12) %	

## Discussion

This study sought to investigate the real-world long-term effect of isCGM on HbA_1c_ in patients with T1D with poor glycaemic control. We found that patients using isCGM experienced clinically significantly lower HbA_1c_ levels, which persisted for the 24-month period after initiation, and our hypothesis is therefore rejected based on the results of this study.

In an observational study by Lameijer et al. [18], a similar but lower sustained HbA_1c_ improvement of 3.5 mmol/mol was observed among 342 patients with type 1 or 2 diabetes in a 24-month period after initiation of isCGM. Selection bias might have occurred in the study as only half of the invited patients accepted a follow-up. Furthermore, HbA_1c_ values were self-reported, which decreases validity. In another observational study by Nathanson et al. [17], a large cohort of Swedish T1D users of isCGM were compared by propensity score matching with a group of T1D without isCGM, and they found an estimated difference in change from baseline HbA_1c_ of -2.5 mmol/mol for patients with baseline HbA_1c_ ≥ 70 mmol/mol. The indications for isCGM were not clear from the work, and whether the included patients received isCGM for indications other than poor glycaemic control is unknown. If HbA_1c_ ≥ 70 mmol/mol was an indication for reimbursement, matching on HbA_1c_ values might be problematic as patients from the control group with HbA_1c_ ≥ 70 mmol/mol who did not receive isCGM are probably not representative of the general population of T1D. In our study, we found a slightly higher estimated difference between T1D-CGM and T1D-NOCGM, which may be caused by the varying study designs. However, the thorough education in the use of isCGM in Region North Denmark might also have affected the results [13, 19].

Several limitations in our study exist. First, the study was not randomized, and the patients initiating isCGM may have been more motivated to handle their own disease, and other interventions could have had the same glucose-lowering effect. Since the indication HbA_1c_ ≥ 70 mmol/mol for reimbursement of isCGM is objective, we believe this selection bias has been minimized though. Another issue with the lack of randomization is that our T1D-NOCGM group is not comparable with the T1D-CGM group. For example, T1D-CGM patients have much higher HbA_1c_ at baseline due to the inclusion criterion of HbA1c ≥ 70 mmol/mol, but it was not possible to use the same criterion on T1D-NOCGM patients, because patients who did not initiate isCGM despite of the indication are *not* representative of T1D patients in general. Another issue with the study design is that other interventions/changes in the treatment of patients with diabetes in Region North Denmark in the same period could influence our results. However, our addition of a control group with follow-up in the same period makes it possible to assess the effect of any other glucose-lowering interventions for T1D patients. In the same line, data were extracted in the period of COVID-19, which may in general have affected the results. Previous published results show improved glycaemic control during the COVID-19 pandemic [20]. Finally, we lack important information, such as, body mass index, insulin dosing, type of CGM device (Freestyle Libre 1 or 2), type of CSII and device use, which could have strengthened our analyses. For example, compared with Libre 1, the Libre 2 device has real-time alarms to warn about hypo- and hyperglycaemia, which could influence glycaemic control. Furthermore, studies have shown that use of different CSII systems lead to significantly different glycaemic control [21]. Adjustment for the exact type of CSII would thus have strengthened our results.

An inherent risk in observational studies is selection bias, and especially matched designs may be prone to selection bias, because the choice of matched variables is subjective or based on available information. Other important factors, such as motivation, are not available in the data and may potentially be confounding factors. We tried to avoid matching to enable observation of all patients with T1D in the region, which we assess as a strength of the study. Another related strength is the high number of patients included and the regionwide setup. Finally, a strength is that the Diabetes Dataplatform used in this study has undergone significant quality control. For example, the disease codes for all patients in the database have been validated by screening patient journals.

In conclusion, our results indicate that patients with T1D in poor glycaemic control in Region North Denmark in general benefitted from using isCGM with a sustained 24-month improvement in HbA_1c_. However, older patients seem to have less effect of isCGM on HbA_1c_. We believe these results are important and should be taken into consideration by decision makers, but the results should be interpreted with care asour study was not randomized.

## Abbreviations

T1D: Type 1 diabetes
isCGM: intermittently scanned continuous glucose monitoring
HbA_1c_: glycated hemaglobin A1c
SD: standard deviation
CSII: continuous subcutaneous insulin infusion
